# Characterizing the functioning of the attentional networks in state and trait anxiety: the role of affective information

**DOI:** 10.3389/fpsyg.2025.1539992

**Published:** 2025-03-20

**Authors:** Antonia Pilar Pacheco-Unguetti, Alberto Acosta, Juan Lupiáñez

**Affiliations:** ^1^Department of Personality, Assessment, and Psychological Treatment, University of Granada, Granada, Spain; ^2^Mind, Brain, and Behavior Research Center (CIMCYC), University of Granada, Granada, Spain; ^3^Department of Experimental Psychology, University of Granada, Granada, Spain

**Keywords:** trait-anxiety, state-anxiety, attentional networks, affective manipulations, alerting network

## Abstract

**Introduction:**

The aim of the present research was to examine the role of affective information in the functioning of attentional networks in individuals with high vs. low trait or state anxiety. Previous studies suggest that anxiety can influence attentional processes, but the impact of affective information, such as alerting emotional stimuli, on these attentional networks remains unclear.

**Methods:**

We conducted a set of experiments using negative or neutral faces as alerting stimuli, presented either alone or together with a neutral sound, within a modified version of the Attention Network Test-Interactions (ANT-I). Additionally, data from previous experiments with similar anxiety groups and without affective manipulations of alertness were analyzed for comparative insights (378 participants in total).

**Results:**

Results showed three main effects on the functioning of attentional networks when affective alerting signals were introduced: (1) the alertness effect is increased, (2) the interference effect is increased, and (3) the orienting effect is decreased. These effects occurred regardless of the valence of the alerting stimuli on a given trial. Importantly, the presence of affective information on some trials eliminated the group differences regarding the functioning of the attentional networks that are usually found when no affective information is presented. Specifically, the larger interference effect commonly observed in individuals with high trait-anxiety and the larger alertness and orienting effects seen in those with high state-anxiety, disappeared when emotional alerting stimuli were included.

**Discussion:**

The findings suggest that emotional information can significantly impact the functioning of attentional networks, particularly in relation to anxiety. The presence of affective stimuli seems to modulate attentional biases associated with anxiety, potentially neutralizing the usual effects seen in individuals with high trait or state anxiety. The role of affective information on the functioning of the attentional networks is discussed within the framework of anxiety and attention.

## 1 Introduction

Our emotion, cognition and action mechanisms are closely connected and organized to ensure both survival and adaptive responses to environmental events (Pessoa, 2008; Phelps, 2006; Todd et al., 2020). The prioritization of relevant information processing at any given moment -particularly when it is threatening- has been critical for survival by enabling effective responses to potential dangers (Blanchette, 2006; Öhman and Mineka, 2001).

Over the past four decades, the influence of emotion on cognitive functions such as perception, memory or decision-making has interested cognitive psychologists (Blaney, 1986; Bower, 1981; Lerner et al., 2015) and neuroscientists (Dolan, 2002, Pessoa, 2008, or Schwarz, 2000). Among these cognitive functions, the impact of emotion on attention has probably been the most extensively researched topic, given the importance of detecting and managing potential threats (for reviews, see Cisler and Koster, 2010; Compton, 2003; Lang et al., 2000; Taylor and Fragopanagos, 2005; and Yiend, 2010). To gain a deeper understanding of human behavior and experience, it is important to bear in mind the reciprocal influences between emotion and cognition, and the role that individual differences and affective information play in this relationship. Investigating the interplay between emotion and cognition is essential for understanding healthy functioning and the vulnerabilities that arise from psychological disorders (e.g., Dolcos et al., 2011, 2020; Izard, 2009).

This paper focuses on the interaction between attention and emotion through the framework of the three attentional networks: alertness, orienting and cognitive control. In particular, we aim to study whether the efficiency of these networks can be modulated by the affective content of alerting signals in people with high vs. low trait and state anxiety.

### 1.1 Attentional networks, anxiety, and affective stimuli

According to Posner’s neurocognitive model of attention (Petersen and Posner, 2012; Posner and Petersen, 1990), the attentional system is organized into three specialized networks: alerting, orienting and executive control. Each network has a different function. The alerting network is responsible for achieving and maintaining an alert state, the orienting network directs attention toward relevant sensory stimuli, and the executive control network is involved in detecting and resolving conflicts among thoughts, feelings or responses. Despite their distinct roles, these networks operate in an interconnected manner, involving different neural areas and neuromodulators, to regulate behavior and adapt it to environmental demands (i.e., Fan et al., 2005; Petersen and Posner, 2012; Posner and Rothbart, 2007). Research shows that the alerting network exerts a negative influence on the executive control network (Fan et al., 2009; Fossella et al., 2002; Posner, 1994) while enhancing the orienting network (Callejas et al., 2005; Fuentes and Campoy, 2008), and the orienting network supports the executive control network (Fan et al., 2009; Ishigami and Klein, 2010).

Fan et al. (2002) developed the Attention Network Test (ANT) to simultaneously assess the functioning of each attentional network by combining a spatial cueing procedure (Posner, 1980) with a flanker task (Eriksen and Eriksen, 1974). After the ANT, numerous adaptations have been developed subsequently (see de Souza Almeida et al., 2021 for a review). For example, Callejas et al. (2004) modified the task to measure the interactions among the networks by incorporating an auditory tone as a warning signal instead of a visual stimulus (ANT for Interactions, or ANT-I). Ishigami and Klein (2010) demonstrated that both ANT and ANT-I provide reliable measures of the three attentional functions, with the ANT-I potentially offering more sensitivity to measure alertness. In fact, both tasks have been widely used in genetic (Fan et al., 2001; Fossella et al., 2002), developmental (Boen et al., 2021; Lewis et al., 2018; Posner et al., 2012), neuroimaging (Fan et al., 2005; Markett et al., 2014; Neuhaus et al., 2010) and clinical studies (Berger and Posner, 2000; Dini et al., 2020; Gao et al., 2023; Miró et al., 2011; Russman Block et al., 2017; Wang et al., 2020).

Identifying individual differences in the efficiency of these networks is important for recognizing specific attentional alterations that may contribute to some pathologies or increase vulnerability to them (Casagrande et al., 2017; Gu et al., 2008; Matthews and Zeidner, 2012; Posner et al., 2002). We have undertaken a series of studies investigating whether anxiety (both trait and state) and anxiety disorders are related to specific or generalized impairments in the attentional networks’ functioning. In studies using the standard version of the ANT-I, which does not include affective manipulations of stimuli, we found that trait-anxiety (TA) was linked to deficiencies in the executive control network. In contrast, state-anxiety (SA) was associated with an over-functioning of the alerting and orienting networks (Pacheco-Unguetti et al., 2010). Additionally, individuals with anxiety disorders exhibited both reduced effectiveness in the executive control network and difficulties in disengaging attention from invalid cues (Pacheco-Unguetti et al., 2011). These findings, along with data from other studies (Bar-Haim et al., 2007; Berggren and Derakshan, 2013, 2014; Cisler and Koster, 2010; Eysenck et al., 2023; Garner et al., 2012; Heeren et al., 2015; Leskin and White, 2007; Reinholdt-Dunne et al., 2013; Tortella-Feliu et al., 2014; Van Bockstaele et al., 2014; Wang et al., 2020), reveal the specific functioning of attention in clinical and subclinical anxious populations.

Several studies have attempted to clarify the effect of emotional manipulation on attentional networks, but the current body of literature does not yet provide consistent findings. For example, Cohen et al. (2011) conducted two experiments using negative and neutral pictures from the International Affective Picture System (IAPS; Lang et al., 2005) in place of the usual orienting cues (asterisks) in the ANT. They did not observe significant interactions between these emotional cues and the orienting and alerting networks. However, contrary to expectations, participants’ responses in congruent trials were delayed compared to incongruent ones when negative pictures were presented. The authors suggested that this overall reduced interference effect after negative cues may occur because emotion modulates attention in the absence of higher cognitive or executive processes. Nevertheless, when a task involves conflict resolution and top-down control is activated (i.e., in incongruent trials), executive processes attenuate the impact of emotional signals, enabling adaptive conflict management.

In another study, Finucane and Power (2010), to induce a state of fear, introduced a fearful vs. neutral IAPS’ picture at the beginning of each ANT trial. Participants were selected based on high vs. low trait fear scores, and both state and trait anxiety levels were checked prior to the task. Results did not reveal differences between the fear groups regarding the functioning of any of the attentional networks. However, the fear induced by the pictures together with the state-anxiety reported by participants, enhanced the executive control network, thus showing reduced interference. The authors pointed out that in fear-eliciting situations the breadth of attention is narrowed, thus improving the inhibition of irrelevant information (i.e., incongruent flankers) to facilitate responses. As in the studies mentioned above and some others (Gómez-Íñiguez et al., 2014; Zhang et al., 2015), they did not find that negative information had a significant effect on orienting and alertness.

These findings shed some further insight into the intriguing reciprocal relationship between emotional and attentional systems, suggesting that the influence of affective stimuli can be either attenuated or increased to ensure a rapid and adaptive resolution of conflict situations. However, the conditions under which this modulation occurs are quite complex; while some studies have shown a general effect, others have linked it to the level of threat. Additionally, enhanced conflict resolution has been demonstrated in the normal population using emotional and neutral words in a version of the flanker task (Kanske and Kotz, 2010, 2011). It remains unclear whether conflict modulation in response to affective stimuli occurs in people with deficits in top-down control or an attentional bias toward emotional information, such as those with high state or trait anxiety. Additionally, research on how affective information modulates alertness and orienting remains inconclusive, indicating the need for further investigation.

### 1.2 Antecedents and current study

In order to explore the role of affective information in the relationship between anxiety and attentional networks, we conducted experiments that manipulated the emotional nature of the alerting signal. We focused on alertness because it is the most basic aspect of attention, essential for fast responses to stimuli in the environment (i.e., Sturm and Willmes, 2001), and it remains uncertain whether its activation can be influenced by affective information. Additionally, alertness interacts with both the orienting and executive control networks (i.e., Callejas et al., 2004; Fan et al., 2009; Ishigami and Klein, 2010; Posner, 1994), enabling us to examine how emotional stimuli may influence all three attentional networks, as it is the first stimulus presented in the ANT-I.

In a first study (Pacheco-Unguetti et al., 2009), we modified the ANT-I by incorporating emotional sounds to manipulate the alerting network in participants with high vs. low trait-anxiety. The results indicated a general enhancement of the alerting effect compared to a previous study without affective stimuli (Pacheco-Unguetti et al., 2010, Exp. 1). Surprisingly, positive, negative and neutral sounds (a woman’s scream, a baby’s laugh and a yawn) produced the same alerting effect and they did so in both high and low trait-anxiety groups. Overall, alerting was increased independently of the emotional content of the signals and anxiety levels. We posited that the social and biological relevance of the selected stimuli might explain these findings, as such stimuli capture attention automatically by default (i.e., Öhman and Mineka, 2001; Yiend, 2010). Thus, people scoring high and low in TA exhibited a higher, but similar, alerting level. However, we did not dismiss the potential influence of other factors, such as the modality of the alerting signal, or even the possibility that the alerting system is only slightly sensitive to affective manipulations. Importantly, the alerting network function is to prepare individuals to process and respond to incoming stimulation, suggesting that its activation inherently involves an intrinsic affective or motivational state. Therefore, it is not necessary for a warning signal to alert us to be relevant for the task or affectively charged.

Building on the results and explanations of the first experiment with sounds, we conducted a series of experiments presenting emotional faces alone (Experiment 1) or combined with the neutral sound from the standard ANT-I (Experiment 2) as alerting stimuli. In each experiment, participants were selected for having high vs. low trait-anxiety scores or received an anxiety vs. positive mood-induction before the tasks, resulting in eight groups with 182 participants. We hypothesized that if the alerting network is not sensitive to these manipulations due to its “intrinsic” affective nature, we would not observe differences in alerting levels between anxiety groups in any of our experiments. However, based on previous findings (Pacheco-Unguetti et al., 2009, 2010), we anticipated impaired executive control in participants with high trait-anxiety, and an over-functioning of the alerting and orienting networks in participants with high state-anxiety. Alternatively, if the emotional nature of the signal increases alertness depending on trait or state anxiety, we might expect a greater alerting effect for negative stimuli (i.e., angry faces) in our high anxiety groups due to their bias toward threatening information (see Bar-Haim et al., 2007). Nevertheless, we did not rule out the possibility that emotional faces might minimize the possible differences between groups, as their relevance could also increase alertness in low-anxiety participants. This means both groups might show a higher alerting level compared to when signals are not affective. Since research on the emotional modulation of the orienting and executive control networks has yielded inconsistent results, we refrained from making specific predictions about how affective alerting signals would influence these networks.

Finally, due to the possibility that affective manipulations could have a general effect on the alerting network and not only a specific, i.e., between trials effect (negative vs. neutral stimuli), we decided to assess the impact of affective alerting signals on the attentional networks. To do so, we compared experiments with and without affective manipulations, by performing an overall analysis comparing the data from the two experiments reported in the current paper with those from previous experiments that used neutral stimuli, involving a total of 378 participants. This analysis is key to understanding how affective signals modulate the effects of anxiety on attentional network functioning.

## 2 Experiment 1: anxiety and affective visual alerting signal

In this experiment, a face was used as a warning signal to replace the warning sound of the standard ANT-I task. In Experiment 1A, trait anxiety was manipulated by selecting participants on the basis of their high or low scores on trait anxiety; whereas, to manipulate state anxiety, in Experiment 1B, participants with medium scores were induced to a high or low anxiety state.

### 2.1 Methods

#### 2.1.1 Participants

Ninety-four psychology students (mean age = 20.02, *SD* = 2.97; 20 males) from the University of Granada (Spain) were selected from 252 incoming first-year students based on their high, medium or low TA scores as measured by the Spanish version of the State–Trait Anxiety Inventory (STAI-TA; Spielberger et al., 1982). In Experiment-1A, 26 participants were in the High Trait-Anxiety group (HTA; ≥80th percentile), and 26 participants were in the Low Trait-Anxiety group (LTA; ≤15th percentile). The remaining 42 students, selected for their medium-to-low STAI-TA scores (percentiles between 25 and 30), were randomly assigned to one of two groups upon arrival for Experiment-1B: the High State-Anxiety group (HSA) who received a negative mood induction, and the Low State-Anxiety group (LSA) who received a positive mood induction (see Table 1 for descriptive statistics). The selection by medium-to-low levels of TA guarantees that the effects were due to the experimentally induced SA rather than any co-varying TA.

**Table 1:** Mean scores and standard deviation (in parentheses) in the STAI-TA (range 0–60), STAI-SA (range 0–60) and Mood Evaluation Subscales of EVEA (range 0–10) for Experiments 1A and 1B.

Experiment-1A		STAI-TA	STAI-SA	EVEA-Anxiety	EVEA-Happiness
High trait anxiety		39.58 (6.26)		4.15 (2.75)	3.59 (2.13)
Low trait anxiety		9.96 (3.18)		1.12 (1.20)	6.47 (2.43)
Experiment-1B					
High state anxiety	PRE	18.10 (8.48)	14.57 (9.23)	1.10 (1.13)	6.39 (2.39)
	POST		26.57 (8.94)	5.43 (1.67)	3.14 (1.86)
Low state anxiety	PRE	17.04 (7.87)	12.50 (3.76)	0.90 (0.68)	7.22 (1.19)
	POST		8.00 (1.71)	0.20 (0.41)	7.40 (1.68)

All participants were selected according to STAI-TA scores (high vs. low for Experiment-1A and medium levels for Experiment-1B). EVEA responses were collected after the experiment in Experiment-1A and before and after the mood induction in Experiment-1B together with the STAI-SA.

All participants were naïve as to the purpose of the experiment and received course credit for their participation. The experiments were conducted in accordance with the ethical guidelines laid down by the University of Granada, in accordance with the ethical standards outlined in the 1964 Declaration of Helsinki (latest revision: Seoul, 2008).

#### 2.1.2 Mood induction

The mood-induction procedure for Experiment-1B included two sets of 10 pictures selected from the IAPS (Lang et al., 2005), based on the Spanish normative data for valence and arousal (Vila et al., 2001). One set contained images with positive emotional content (i.e., couples, families or landscapes), while the other set featured negative images (i.e., mutilations, victims of violence or natural disasters). Each picture was displayed for 6 s and was always preceded by a brief text alluding to the content, which was also shown for 6 s before the image and remained on screen during its presentation.

Given that the images selected according to the aforementioned criteria varied in the types of scenes depicted, they were accompanied by brief texts that added specific nuances to the induced emotion. For the anxiety induction, accompanying texts emphasized characteristics inherent to anxiety, such as uncertainty, lack of control over circumstances or events, and the anticipation of imminent dangers or threats (e.g., a baby with a tumor on his eye with the text “*Health is a valuable good, but diseases can arise unexpectedly in ourselves or our loved ones”*). In the positive mood induction, the texts focused on life’s opportunities and achieving goals (e.g., an image of a medal ceremony with the text “*When we achieve our goals, we feel empowered. There are always personal accomplishments in our lives”*). This mood-induction procedure has been developed in our laboratory and successfully used in previous studies at both individual and group levels (e.g., Pacheco-Unguetti et al., 2010, 2012, 2014).

#### 2.1.3 Stimuli

We used faces of 3 men from the *Facial Action Coding System* (FACS; Ekman and Friesen, 1978) as visual alerting signals. Each face displayed either an angry or neutral expression (FACS codes: JB1-23, JB1-3; PE2-21, PE2-4; WF3-4, WF2-5). The size of each image was 4.5 × 7.5 cm. An asterisk, presented 0.6° of visual angle above or below fixation point, was used as an orienting signal. The target consisted of a central arrow (length 0.55°) that could point either to the right or left, together with two additional arrows on each side (0.06° away) pointing in the same or opposite direction.

#### 2.1.4 Task

**Figure 1:**
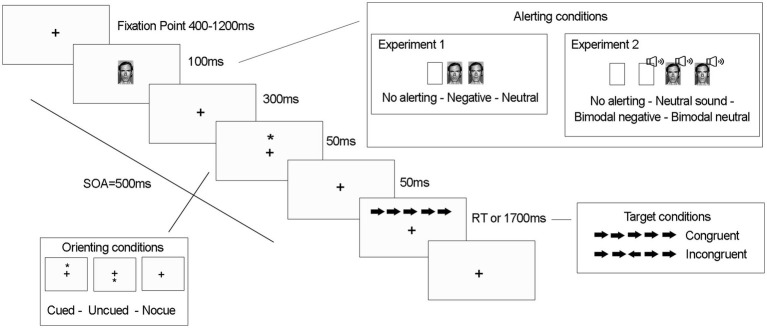
Sequence of events for each trial and stimuli conditions of the experiments 1 and 2.

The sequence of events for each trial is illustrated in Figure 1. Each trial started with a fixation cross presented in the center of the screen for 400–1,200 ms. In 2/3 of the trials, the alerting signal (face) was presented for 100 ms (half had neutral valence and half were negative). In the remaining 1/3 of trials the signal was not presented. After 400 ms, for 2/3 of the trials, an asterisk was presented for 50 ms as an orienting signal either above or below the fixation point (no asterisk was presented in the remaining third). Then, the asterisk disappeared, leaving only the fixation point. After 50 ms, an arrow flanked by two distracting arrows on each side was presented for 1700 ms or until the participant’s response, in the same location as the previous orienting cue (cued trials) or in the opposite location (uncued trials). The distracters could be pointing either in the same direction as the target arrow (congruent trial) or in the opposite direction (incongruent trial). Participants had to discriminate the direction of the central arrow by pressing as quickly as possible either “c” (for leftward direction) or “m” (for rightward direction) while ignoring the flanking arrows.

Each participant completed a practice block of 20 trials followed by 4 blocks of 72 trials (these were presented randomly) with a break to rest between blocks.

#### 2.1.5 Procedure

Participants were tested individually in a dimly lit cubicle, in one session that lasted for approximately 50 min. Upon arrival at the laboratory, they were briefly informed about the general procedure of the study and completed the consent form.

Participants in Experiment-1A directly carried out the experimental task. They read the task instructions on the screen, which were emphasized by the experimenter, and asked any questions for clarification before the beginning of the experiment. After completing the task, participants filled out the Mood Evaluation Scale (EVEA; Sanz, 2001; see Sanz et al., 2014 for a review of the psychometric properties of the scale), in order to check whether the participants’ mood after the task could be affecting the results or whether these were only due to the TA variable. The EVEA is a scale containing 16 items (ranging from 0 to 10), which offers a measure of 4 mood states (Anxiety, Hostility, Depression, and Happiness). Given that state-anxiety co-varies with trait-anxiety, and the mood induction procedure specifically enhanced a negative affective state related to anxiety, only the Anxiety subscale was considered for further analysis (variations in the Hostility and Depression subscales were less pronounced and therefore excluded). The Happiness subscale was also included, as the mood induction procedure included a positive condition.

Participants in Experiment-1B were randomly assigned to one of two groups on arrival to the testing site. To check the effectiveness of the mood induction, they filled out the State-Anxiety subscale of the STAI (STAI-SA) and the EVEA immediately before and after the procedure. Once the tests were filled out for the first time, participants were informed that they would see a series of pleasant or unpleasant pictures (depending on the group they were assigned to) and their task would be to read the sentences, look at the pictures and try to involve themselves emotionally in the content. After the mood induction, participants filled out the questionnaires again and then performed the experimental task as participants in Experiment-1A.

#### 2.1.6 Data analysis

Behavioral data were analyzed as in previous studies (Callejas et al., 2004, 2005; Pacheco-Unguetti et al., 2009, 2010, 2011). After performing a general analysis to confirm that the task worked in the usual way, we computed an efficiency index for each attentional network with the following RT subtractions: *Alerting* = No alerting – Alerting signal (negative and neutral faces), restricted to the no-cue condition; *Orienting* = Uncued - Cued; *Executive* control = Incongruent – Congruent. We carried out mixed ANCOVAs with group as a between-groups factor and the variable network (functional index for each attentional network) as a within-participant variable. State-anxiety score on the EVEA was introduced as a covariate in Experiment-1A, and STAI-TA scores in experiment-1B.

Trials with incorrect responses (2.89%) or with extreme values (RTs smaller than 200 ms or higher than 1,500 ms; 0.97%) and data of 3 participants (2 of the HTA group in Experiment-1 and 1 of the HSA group in Experiment-2) with more than 50% errors were excluded from the analyses.

### 2.2 Results

#### 2.2.1 Experiment-1A: trait-anxiety

##### 2.2.1.1 Self-report questionnaire scores analysis

A unifactorial ANOVA was carried out with the STAI-TA scores (selection criteria) of both groups as the dependent variable. As we expected, the main effect of group was significant [*F*(1, 48) = 454.38, *MSE* = 24.1, *p* < 0.001, $\eta_{p}^{2}$ = 0.90], showing greater anxiety level in the HTA than in the LTA group. The same analysis was performed for the Anxiety and Happiness EVEA subscales scores obtained after the experiment (see Table 1). The HTA showed significantly higher levels than the LTA group in Anxiety [*F*(1, 48) = 26.11, *MSE* = 4.39, *p* < 0.001, $\eta_{p}^{2}$ = 0.35]; however, the LTA showed greater levels than the HTA group in Happiness [*F*(1, 48) = 19.64, *MSE* = 5.26, *p* < 0.001, $\eta_{p}^{2}$ = 0.29].

##### 2.2.1.2 General analysis

Mean RTs for correct responses per experimental condition were introduced into a 2 (group) × 3 (alerting) × 3 (orienting) × 2 (congruency) factorial mixed ANOVA. The main effect of each within-participant variable was significant, showing the expected effects of alerting [*F*(2, 96) = 50.02, *MSE* = 1941, *p* < 0.001, $\eta_{p}^{2}$ = 0.51], orienting [*F*(2, 96) = 157.35, *MSE* = 1,499, *p* < 0.001, $\eta_{p}^{2}$ = 0.76], and congruency [*F*(1, 48) = 241.35, *MSE* = 9,013, *p* < 0.001, $\eta_{p}^{2}$ = 0.83]. In addition, the usual interactions were also observed: alerting × orienting [*F*(4, 192) = 10.62, *MSE* = 970, *p* < 0.001, $\eta_{p}^{2}$ = 0.18], alerting × congruency [*F*(2, 96) = 4.98, *MSE* = 958, *p* = 0.008, $\eta_{p}^{2}$ = 0.09], orienting × congruency [*F*(2, 96) = 20.32, *MSE* = 1,126, *p* < 0.001, $\eta_{p}^{2}$ = 0.29], and alerting × orienting × congruency [*F*(4, 192) = 3.97, *MSE* = 1,041, *p* = 0.004, $\eta_{p}^{2}$ = 0.07]. All these main effects and interactions showed the pattern usually observed with this task (Callejas et al., 2004; Pacheco-Unguetti et al., 2009, 2010, 2011). However, none of these main effects or interactions was modulated by group.

Regarding alertness, there was no difference between negative and neutral alerting signals (*F* < 1), although both led to faster RTs than the no-alerting signal condition: negative vs. no-alerting [*F*(1, 48) = 71.38, *MSE* = 2097, *p* < 0.001, $\eta_{p}^{2}$ = 0.59]; neutral vs. no-alerting [*F*(1, 48) = 51.18, *MSE* = 2,762, *p* < 0.001, $\eta_{p}^{2}$ = 0.51]. More importantly, when the same ANOVA was repeated but excluding the no-alerting condition, the emotionality of the alerting stimuli did not show a significant main effect, and the significant interactions between alerting and the other variables completely disappeared (all *Fs <* 1). In other words, the usual alerting effect was significantly observed and modulated both the orienting and the executive control networks, but the neutral and negative alerting cues were equally effective in alerting the system.

##### 2.2.1.3 Attentional index analysis

We subsequently carried out an ANCOVA with group as a between-groups factor, the variable network (functional index for each attentional network) as a within-participants variable, and the state-anxiety score on the EVEA as a covariate. The interaction group × network was significant [*F*(2, 94) = 4.60, *MSE* = 1,333, *p* = 0.012, $\eta_{p}^{2}$ = 0.09], indicating that although groups did not differ in the executive control [*F*(1, 47) = 2.87, *MSE* = 1945, *p* = 0.096, $\eta_{p}^{2}$ = 0.05], and orienting indices (*F* < 1), they did differ in the alerting index [*F*(1, 47) = 4.13, *MSE* = 1,553, *p* = 0.047, $\eta_{p}^{2}$ = 0.08]. The HTA group had a lower alerting index than the LTA group (see Table 2).

**Table 2:** Attentional indexes of executive control, orienting and alerting (negative and neutral face cues) for each group and Experiments 1A and 1B.

Experiment-1A	Executive control	Orienting	Alerting	Negative alerting	Neutral alerting
High trait anxiety	104.40	60.60	33.96	33.83	34.09
Low trait anxiety	92.41	51.63	56.71	58.97	54.45
Experiment-1B					
High state anxiety	107.29	49.92	63.54	60.95	62.23
Low state anxiety	92.62	50.07	51.80	50.81	53.25

#### 2.2.2 Experiment-1B: state-anxiety

##### 2.2.2.1 Self-report questionnaire scores analysis

STAI-SA scores taken before and after the mood induction were entered into a 2 (group: HSA, LSA) × 2 (time: before, after induction) mixed ANOVA. The key result was a significant interaction between group and time [*F*(1, 39) = 86.49, *MSE* = 16, *p* < 0.001, $\eta_{p}^{2}$ = 0.69]. As expected, STAI-SA scores were similar for both groups before induction (*F* < 1), but were significantly higher in the HSA compared to the LSA group following induction [*F*(1, 39) = 91.33, *MSE* = 38.53, *p* < 0.001, $\eta_{p}^{2}$ = 0.70] (see Table 1).

A similar analysis was performed on the scores for the anxiety and happiness EVEA subscales. The interaction between group and time was significant in both subscales (*p*s < 0.001). Anxiety scores were similar for both groups before induction (*F* < 1), but scores were significantly higher in the HSA compared to the LSA group after induction, *F* (1, 39) = 201.62, *MSE* = 1.38, *p* < 0.001, $\eta_{p}^{2}$ = 0.83. The opposite pattern of results was observed for the happiness subscale. Scores were similar for both groups before induction, *F* (1, 39) = 2.07, *MSE* = 3.40, *p* = 0.157, $\eta_{p}^{2}$ = 0.05, but diminishing significantly in the HSA group compared to the LSA group after induction, *F* (1, 39) = 59.21, *MSE* = 3.13, *p* < 0.001, $\eta_{p}^{2}$ = 0.60.

##### 2.2.2.2 General analysis

Similar analyses to those performed in Experiment-1A were carried out, yielding comparable results regarding the main effects of each within-participants variable and their interactions. The main effect of alerting [*F*(2, 78) = 135.04, *MSE* = 775, *p* < 0.001, $\eta_{p}^{2}$ = 0.77], orienting [*F*(2, 78) = 145.85, *MSE* = 1,078, *p* < 0.001, $\eta_{p}^{2}$ = 0.79], and congruency [*F*(1, 39) = 278.26, *MSE* = 6,504, *p* < 0.001, $\eta_{p}^{2}$ = 0.87], was significant, as well as the interactions alerting × orienting [*F*(4, 156) = 17.91, *MSE* = 572, *p* < 0.001, $\eta_{p}^{2}$ = 0.31], alerting × congruency [*F*(2, 78) = 9.10, *MSE* = 663, *p* < 0.001, $\eta_{p}^{2}$ = 0.19], orienting × congruency [*F*(2, 78) = 37.41, *MSE* = 603, *p* < 0.001, $\eta_{p}^{2}$ = 0.49], and alerting × orienting × congruency [*F*(4, 156) = 3.71, *MSE* = 623, *p* = 0.006, $\eta_{p}^{2}$ = 0.08]. Importantly, as in Experiment-1A, none of these main effects or interactions was modulated by group.

Once again, there was no difference between negative and neutral alerting signals (*F* < 1), although both led to faster RTs than the no-alerting signal condition: negative vs. no-alerting [*F*(1, 39) = 159.60, *MSE* = 961, *p* = < 0.001, $\eta_{p}^{2}$ = 0.80]; and neutral vs. no-alerting [*F*(1, 39) = 173.66, *MSE* = 924, *p* = < 0.001, $\eta_{p}^{2}$ = 0.81]. The ANOVA excluding the no-alerting trials did not show a significant main effect of the emotionality of the alerting stimuli (*F <* 1), and the interactions of alerting with orienting (*F <* 1) and congruency [*F*(1, 39) = 1.42, *MSE* = 546, *p* = 0.240, $\eta_{p}^{2}$ = 0.03], disappeared as in Experiment-1A.

##### 2.2.2.3 Attentional index analysis

The ANCOVA with group as a between-groups factor, the variable network as a within-participants variable and STAI-TA scores as a covariate showed that the interaction group *×* network was not significant (*F* < 1). Groups did not differ on the executive control [*F*(1, 38) = 1.74, *MSE* = 1227.83, *p* = 0.195, $\eta_{p}^{2}$ = 0.04], orienting (*F* < 1), or alerting indices [*F*(1, 38) = 1.73, *MSE* = 537.96, *p* = 0.195, $\eta_{p}^{2}$ = 0.04].

### 2.3 Discussion

The results of Experiment-1A showed that the TA groups differed significantly only in the alerting network functioning. Contrary to expectations, the HTA group had a lower alerting index than the LTA group, although this difference was not influenced by facial expression valence. Specifically, both groups showed faster RTs in conditions where neutral or angry faces were presented compared to no-alerting conditions. It is worth noting that the HTA showed a similar level of alerting (approximately 30 ms) to that found with the original version of the task and in our previous study with non-affective alerting signals in individuals with TA (Callejas et al., 2005; Pacheco-Unguetti et al., 2010, Experiment-1). Therefore, it was the LTA group that significantly increased the alerting level (about 59 ms), regardless of whether the stimuli were neutral or negative. Apart from this, no significant differences between groups were found in the functioning of the orienting network. In the executive control, the HTA group showed only a trend toward a greater level of interference.

In Experiment-1B, no significant differences were observed between groups in the functioning of the attentional networks. The alerting index increased equally in both HSA and LSA groups (61 vs. 52 ms) compared to the original task.

Overall, these findings suggest that the general alerting level can be enhanced by warning signals more complex than a neutral tone, like a human face. Nevertheless, the valence of faces did not influence the alerting network functioning. However, the greater interference effect found for the TA groups in previous studies without emotional stimuli (Pacheco-Unguetti et al., 2010, Experiment-1) seems to be diminished by this alerting manipulation (the difference between groups did not reach significance, as with emotional sounds in the study by Pacheco-Unguetti et al., 2009). The same applies to the over-functioning of the alerting and orienting networks previously reported in individuals with high SA (Pacheco-Unguetti et al., 2010, Experiment-2), as our state-anxiety groups did not differ in alertness and orienting when the emotionality of the stimuli was manipulated.

## 3 Experiment 2: anxiety and affective bimodal manipulation of the alerting network

However, before extracting solid conditions, we decided to replicate the study. Furthermore, since in daily life it is quite common that stimuli from different modalities co-occur and serve as alerting cues, we found it worthwhile to examine the effects of affective bimodal signals on the functioning of attentional networks. Most studies using bimodal cues have been more focused on spatial attention than alertness (i.e., Koelewijn et al., 2010; Zimmer et al., 2022), and generally they did not reach decisive conclusions about the differing effects that each kind of stimulus can produce. Some studies have shown cumulative effects of bimodal cues compared to unimodal ones (i.e., Stanford and Stein, 2007), but some others have reported comparable effects (i.e., Santangelo et al., 2008; Spence and Driver, 1999). We have shown that using auditory or visual affective stimuli enhances alertness compared to a neutral tone. This improvement in alertness also seemed to reduce differences between anxious groups found in previous studies. Therefore, it is important to explore the effect of affective bimodal cues in the ANT-I. If these cues have additive effects, we expect to increase the effect of these signals on the alerting network and thus reduce even more the small differences observed between groups in the functioning of attentional networks.

### 3.1 Methods

#### 3.1.1 Participants

Eighty-eight psychology students (mean age = 20.04, SD = 4.68; 11 males) from the University of Granada (Spain) were selected using the same selection criteria as in Experiment-1. Twenty participants were in the HTA group (≥80th percentile), and 20 participants (≤15th percentile) made up the LTA group (Experiment-2A). Forty-eight participants with medium-to-low levels on the STAI-TA (percentiles between 30 and 35) were selected and randomly assigned to HAS or LSA group on arrival at the testing site (Experiment-2B).

#### 3.1.2 Mood induction materials, stimuli, task and procedure

The mood induction materials and procedure for this experiment were similar to those used in Experiment-1, with only two changes. First, we included an alerting tone (2.000 Hz, 50 ms) accompanying the two faces (bimodal alerting conditions) and presented alone in another condition (see Figure 1). Due to the addition of this new alerting condition, the number of trials per block increased and each participant carried out 4 blocks of 96 randomized trials. Second, to avoid extending the experiment beyond 1 h, and since mood-induction procedures have been successfully used in previous studies (e.g., Pacheco-Unguetti et al., 2010, 2012) and also in Experiment-1B, we decided to administer the STAI-SA and EVEA questionnaires only after the experimental task (Experiment-2A) and after the mood induction (Experiment-2B).

#### 3.1.3 Data analysis

Behavioral data were analyzed as in Experiment 1. Trials with incorrect responses (1.98%) or with extreme values (RTs smaller than 200 ms or higher than 1,500 ms; 0.43%) were excluded from the analyses.

### 3.2 Results

#### 3.2.1 Experiment-2A: trait anxiety

##### 3.2.1.1 Self-report questionnaire scores analysis

A unifactorial ANOVA was conducted with the STAI-TA scores (selection criteria) of both groups as the dependent variable (see Table 3). As expected, the main effect of group was significant [*F*(1, 37) = 533.60, *p* < 0.001, $\eta_{p}^{2}$ = 0.93], showing greater anxiety level in the HTA group compared to the LTA group. The same analysis was performed with the STAI-SA scores obtained after the experiment, showing significantly greater levels in the HTA compared to the LTA group [*F*(1, 37) = 48.13, *MSE* = 91.02, *p* < 0.001. $\eta_{p}^{2}$ = 0.56]. The analysis performed for the EVEA subscales scores obtained after the experiment yielded results like those obtained in Experiment-1. The HTA group showed significantly higher levels than the LTA group in anxiety [*F*(1, 37) = 23.24, *MSE* = 3.63, *p* < 0.001, $\eta_{p}^{2}$ = 0.38], and the LTA group showed greater levels than the HTA group in the happiness subscale [*F*(1, 37) = 26.48, *MSE* = 5.29, *p* < 0.001, $\eta_{p}^{2}$ = 0.41].

**Table 3:** Mean scores and standard deviation (in parentheses) in the STAI-TA, STAI-SA and Mood Evaluation Subscales of EVEA for experiments 2A and 2B.

Experiment-2A	STAI-TA	STAI- SA	EVEA-Anxiety	EVEA-Happiness
High trait anxiety	40.63 (4.82)	30.10 (12.24)	4.89 (1.95)	2.90 (2.22)
Low trait anxiety	8.55 (3.81)	8.90 (5.93)	1.95 (1.86)	6.70 (2.36)
High state anxiety	19.95 (1.87)	32.25 (8.53)	5.15 (2.36)	3.71 (2.18)
Low state anxiety	19.83 (1.68)	8.25 (5.12)	0.69 (0.79)	7.17 (2.07)

All participants were selected according to STAI-TA scores (high vs. low for Exp 2A and medium levels for Exp 2B). STAI-SA and EVEA responses were collected after the experiment in Exp 2A and after the mood induction in Exp 2B.

##### 3.2.1.2 General analysis

RTs analyses similar to those performed in Experiment-1 were performed, but this time the alerting variable had 4 levels: tone, no-alerting, bimodal negative and bimodal neutral. The ANOVA including all conditions showed as significant the main effects of alerting [*F*(3, 111) = 65.77, *MSE* = 1,515, *p* < 0.001, $\eta_{p}^{2}$ = 0.64] orienting [*F*(2, 74) = 91.29, *MSE* = 1,293, *p* < 0.001, $\eta_{p}^{2}$ = 0.71], and congruency [*F*(1, 37) = 364.58, *MSE* = 7,103, *p* < 0.001, $\eta_{p}^{2}$ = 0.91], and the interactions alerting × orienting, [*F*(6, 222) = 14.36, *MSE* = 675, *p* < 0.001, $\eta_{p}^{2}$ = 0.28], alerting × congruency [*F*(3, 111) = 31.24, *MSE* = 641, *p* < 0.001, $\eta_{p}^{2}$ = 0.46], orienting × congruency [*F*(2, 74) = 14.49, *MSE* = 736, *p* < 0.001, $\eta_{p}^{2}$ = 0.28], and alerting × orienting × congruency [*F*(6, 222) = 2.99, *MSE* = 470, *p* = 0.007, $\eta_{p}^{2}$ = 0.07]. As in the Experiment-1, the factor group did not modulate any interaction or main effect.

Regarding alertness, there was no difference between the bimodal negative and bimodal neutral alerting signals (*F* < 1). Both conditions produced faster RTs than the no-alerting signal condition [bimodal negative vs. no-alerting *F*(1, 37) = 95.69, *MSE* = 2,246, *p* < 0.001, $\eta_{p}^{2}$ = 0.72]; bimodal neutral vs. no-alerting [*F*(1, 37) = 84.22, *MSE* = 2,694, *p* < 0.001, $\eta_{p}^{2}$ = 0.69]. Also, both bimodal signals led to faster RTs than the unimodal tone signal condition [bimodal negative vs. unimodal tone *F*(1, 37) = 52.55, *MSE* = 791, *p* < 0.001, $\eta_{p}^{2}$ = 0.58]; bimodal neutral vs. unimodal tone [*F*(1, 37) = 64.38, *MSE* = 729, *p* < 0.001, $\eta_{p}^{2}$ = 0.63], and the unimodal tone signal produced faster RTs than the no-alerting signal condition [*F*(1, 37) = 34.43, *MSE* = 1959, *p* < 0.001, $\eta_{p}^{2}$ = 0.48]. As in the Experiment-1, the analyses including only the trials where the emotionality of the alerting signal was manipulated (i.e., bimodal negative and bimodal neutral) did not show a significant main effect of alerting, and the interactions of alerting with orienting and congruency disappeared (all *Fs <* 1).

##### 3.2.1.3 Attentional index analysis

The ANCOVA with group as a between-groups factor, the variable network as a within-participants variable and the state-anxiety score on the EVEA as a covariate (see Table 4) did not show as significant the group *×* network interaction (*F <* 1). The HTA and LTA groups did not differ on any of the attentional indices (all *Fs* < 1).

**Table 4:** Attentional indexes of executive control, orienting, and alerting (negative bimodal, neutral bimodal, tone, and no alerting cues) for each group and Experiments 2A and 2B.

Experiment-2A	Executive control	Orienting	Alerting	Bimodal negative alerting	Bimodal neutral alerting	Unimodal tone alerting
High trait anxiety	108.27	40.74	58.19	60.78	60.34	53.45
Low trait anxiety	102.18	36.85	51.99	55.94	54.18	45.86
Experiment-2B						
High state anxiety	91.76	44.36	61.55	66.69	68.35	49.62
Low state anxiety	102.69	42.98	63.42	65.55	68.47	56.24

#### 3.2.2 Experiment-2B: state anxiety

##### 3.2.2.1 Self-report questionnaire scores analysis

A unifactorial ANOVA with group as the independent variable was performed for STAI-SA scores and each EVEA subscale, contrasting the measures taken after the mood induction. As expected, STAI-SA postlevels were significantly higher in the HSA than in the LSA group [*F*(1, 46) = 139.39, *MSE* = 49.59, *p* < 0.001, $\eta_{p}^{2}$ = 0.75] (see Table 3). The same analyses with the anxiety EVEA subscale showed significantly higher scores in the HSA than in the LSA group [*F*(1, 46) = 76.80, *MSE* = 3.10, *p* < 0.001, $\eta_{p}^{2}$ = 0.62]. By contrast, LSA participants showed significantly greater levels than HSA in the happiness subscale [*F*(1, 46) = 31.53, *MSE* = 4.55, *p* < 0.001, $\eta_{p}^{2}$ = 0.40].

##### 3.2.2.2 General analysis

RTs analyses similar to those performed in Experiment-2A were carried out. The ANOVA including all conditions showed as significant the main effects of alerting [*F*(3, 138) = 99.65, *MSE* = 1,452, *p* < 0.001, $\eta_{p}^{2}$ = 0.68], orienting [*F*(2, 92) = 162.64, *MSE* = 1,128, *p* < 0.001, $\eta_{p}^{2}$ = 0.78], and congruency [*F*(1, 46) = 558.01, *MSE* = 4,879, *p* < 0.001, $\eta_{p}^{2}$ = 0.92], and the interactions alerting × orienting [*F*(6, 276) = 12.26, *MSE* = 667, *p* < 0.001, $\eta_{p}^{2}$ = 0.21], alerting × congruency [*F*(3, 138) = 28.21, *MSE* = 670, *p* < 0.001, $\eta_{p}^{2}$ = 0.38], orienting × congruency [*F*(2, 92) = 26.58, *MSE* = 582, *p* < 0.001, $\eta_{p}^{2}$ = 0.36], and alerting × orienting × congruency [*F*(6, 276) = 2.88, *MSE* = 823, *p* = 0.009, $\eta_{p}^{2}$ = 0.06]. As in the Experiment-1, no-group-related interaction effects were observed.

Regarding alertness, there was no significant difference between bimodal negative and bimodal neutral alerting signals (*F* < 1). Both types of signals produced faster RTs than the no-alerting signal condition [bimodal negative vs. no-alerting *F*(1, 46) = 128.69, *MSE* = 2,355, *p* < 0.001, $\eta_{p}^{2}$ = 0.73]; bimodal neutral vs. no-alerting [*F*(1, 46) = 159.00, *MSE* = 2,179, *p* < 0.001, $\eta_{p}^{2}$ = 0.77]. Additionally, both bimodal signals led to faster RTs than the unimodal tone signal condition [bimodal negative vs. unimodal tone *F*(1, 46) = 31.79, *MSE* = 832, *p* < 0.001, $\eta_{p}^{2}$ = 0.41]; bimodal neutral vs. unimodal tone [*F*(1, 46) = 57.47, *MSE* = 701, *p* < 0.001, $\eta_{p}^{2}$ = 0.55], and the unimodal tone signal produced faster RTs than the no-alerting signal condition [*F*(1, 46) = 76.70, *MSE* = 1962, *p* < 0.001, $\eta_{p}^{2}$ = 0.62]. As in all previous experiments, analyses including only the trials where the emotionality of the alerting signal was manipulated (i.e., bimodal negative and bimodal neutral) did not show a significant main effect of alerting, and the significant interactions of alerting with orienting and congruency disappeared (all *Fs <* 1).

##### 3.2.2.3 Attentional index analysis

The ANCOVA with group as a between-groups factor, network as a within-participants variable, and STAI-TA scores as a covariate (see Table 4), did not reveal a significant group × network interaction (F < 1). Both groups had similar orienting, alerting (both Fs < 1), and executive control indices, *F* (1, 45) = 1.98, *MSE* = 785.60, *p* = 0.166, $\eta_{p}^{2}$ = 0.04.

#### 3.2.3 Discussion

The results of Experiment-2 are unambiguous: when affective manipulations of alerting signals were introduced, there was no difference in the functioning of the three attentional networks between the high and low TA and SA groups. Once again, no significant differences were observed regarding the affective valence of the alerting signals or the performance of the attentional networks.

In the Experiment-2A, in contrast to the findings of Experiment-1A, bimodal alerting stimuli increased the alerting level in the HTA group. Consequently, the alerting level in the HTA was not significantly different from the LTA group (59 vs. 50 ms, respectively). Importantly, in Experiment-1A we still observed a trend toward a greater interference in the HTA group consistent with previous studies, however this group showed lower alertness. In the current experiment, probably due to the increase in alerting for the HTA group, the differences in executive control between HTA and LTA groups completely disappeared.

## 4 Comparisons between experiments with and without emotional alerting signals

To further examine whether the HTA participants were enhancing their control or whether the LTA participants were impoverishing it specifically due to the alerting signals, we conducted additional analyses comparing the results of the experiments reported in this paper with findings from our previous studies using the ANT-I. We distinguished between those studies without any affective manipulation (only emotionally neutral stimuli) and those that included some affective manipulation in the experimental task. A total of 378 participants were included in the analyses, combining groups with high vs. low TA and SA.^1^

### 4.1 Experiments with high vs. low trait-anxiety

We conducted an ANCOVA with group (HTA vs. LTA) and affective manipulation (present vs. absent) as categorical factor, the variable network as a within-participants variable, and the SA score from the EVEA as a covariate. Data from 228 participants were included in this analysis.

Three interactions derived from this analysis are worth highlighting. Firstly, the group × network interaction was significant [*F*(2, 452) = 7.08, *MSE* = 854, *p* < 0.001, $\eta_{p}^{2}$ = 0.03], showing the expected larger interference in the HTA group than in the LTA group [*F*(1, 226) = 12.76, *MSE* = 1122.20, *p* < 0.001, $\eta_{p}^{2}$ = 0.05]. The groups did not differ in their functioning of the orienting and alerting networks [*F* < 1 and *F*(1, 226) = 1.11, *MSE* = 944.80, *p* = 0.293, $\eta_{p}^{2}$ = 0.00, respectively]. These results are consistent with those reported in Pacheco-Unguetti et al. (2010; Experiment-1).

Secondly, the affective manipulation × network interaction was significant [*F*(2, 452) = 7.41, *MSE* = 854, *p* < 0.001, $\eta_{p}^{2}$ = 0.03]. When the emotionality of stimuli was manipulated, the alerting index was greater than when emotionality was not manipulated (51 vs. 34 ms) [*F*(1, 226) = 17.65, *MSE* = 944.80, *p* < 0.001, $\eta_{p}^{2}$ = 0.07]. A similar pattern was observed for the interference index (affective signals = 97 ms; neutral signals = 83 ms) [*F*(1, 226) = 9.74, *MSE* = 1122.20, *p* = 0.002, $\eta_{p}^{2}$ = 0.04]. However, the orienting network index remained unchanged regardless of the emotionality manipulations (*F* < 1).

**Figure 2:**
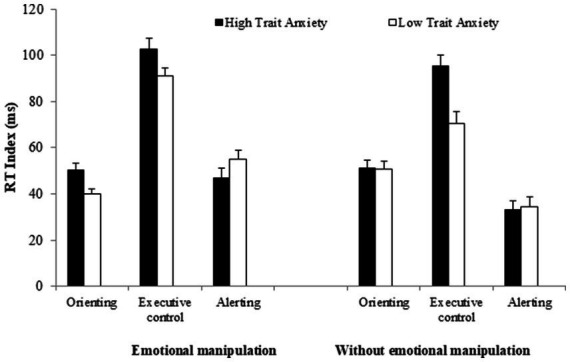
Reaction time (RT) indices of the three attentional networks as a function of participant group (high vs. low trait-anxiety) for experiments with and without emotional manipulations of the alerting signal. Error bars represent standard errors of the mean.

Thirdly, the group × affective manipulation interaction was not significant [*F*(1, 226) = 1.45, *MSE* = 831, *p* = 0.228, $\eta_{p}^{2}$ = 0.00], nor was the group × affective manipulation × network interaction [*F*(2, 452) = 1.27, *MSE* = 854, *p* = 0.281, $\eta_{p}^{2}$ = 0.00]. As shown in Figure 2, when the emotionality of stimuli is manipulated, both groups (particularly the LTA group) exhibit an increased effect of interference. The LTA group shows significantly greater interference with affective manipulation compared to when there is no affective manipulation (93 vs. 73 ms) [*F*(1, 115) = 11.13, *MSE* = 917.2, *p* = 0.001, $\eta_{p}^{2}$ = 0.08]. In contrast, the interference in the HTA group increases only slightly and it is not statistically significant when emotionality of the stimuli is manipulated compared to when it is not manipulated (100 vs. 93 ms) [*F*(1, 110) = 1.05, *MSE* = 1,334, *p* = 0.306, $\eta_{p}^{2}$ = 0.00]. Therefore it is important to note that while both groups show similar interference when emotionality is manipulated [*F*(1, 131) = 1.85, *MSE* = 1,307, *p* = 0.176, $\eta_{p}^{2}$ = 0.01], the interference is significantly greater in the HTA than in the LTA group when it is not manipulated [*F*(1, 94) = 14.53, *MSE* = 874.2, *p* < 0.001, $\eta_{p}^{2}$ = 0.13].

It is important to note that a larger alertness effect is observed in both HTA and LTA groups, when the emotionality of alerting signals is manipulated (left panel) compared to when it is not manipulated (right panel). In the HTA group, the alerting network effect is slightly higher with affective alerting signals than with neutral (47 vs. 34 ms) [*F*(1, 110) = 4.54, *MSE* = 1037.30, *p* = 0.035, $\eta_{p}^{2}$ = 0.04]. In the LTA group, the difference in alertness depending on emotionality manipulations is also significant (affective signals = 54 ms; neutral signals = 33 ms) [*F*(1, 115) = 12.76, *MSE* = 852.60, *p* < 0.001, $\eta_{p}^{2}$ = 0.09]. However, the HTA and LTA groups did no show differences in the alerting level in the experiments with affective alerting signals (HTA = 47 ms; LTA = 54 ms) [*F*(1, 131) = 2.96, *MSE* = 1182.81, *p* = 0.087, $\eta_{p}^{2}$ = 0.02], or in experiments with neutral alerting signals (HTA = 34 ms; LTA = 33 ms; *F* < 1).

In contrast, although the orienting effect is reduced when the emotionality of alerting signals is manipulated (left panel), it does not significantly depend on the emotionality manipulations (*F* < 1). In both, the LTA group [*F*(1, 115) = 2.04, *MSE* = 366.40, *p* = 0.155, $\eta_{p}^{2}$ = 0.02], (affective signals = 46 ms; neutral signals = 51 ms), and the HTA group, *F* < 1, (50 ms in both conditions), the orienting index was similar under affective and neutral conditions. Consequently, we did not find differences between groups in the orienting network scores under either emotional or neutral alerting conditions (both *Fs* < 1).

### 4.2 Experiments with high vs. low state-anxiety

The same analysis was performed for the experiments in which SA was manipulated, resulting in the HSA and LSA groups. In these analyses, STAI-TA scores were used as a covariate. Data from 150 participants were included in this analysis.

Although the group × network interaction did not reach significance [*F*(2, 296) = 1.68, *MSE* = 677.57, *p* = 0.187, $\eta_{p}^{2}$ = 0.01], the groups exhibited significant differences in the functioning of the orienting network [*F*(1, 148) = 7.11, *MSE* = 459.10, *p* = 0.008, $\eta_{p}^{2}$ = 0.04], and a trend toward a difference in alerting [*F*(1, 148) = 3.04, *MSE* = 613.94, *p* = 0.082, $\eta_{p}^{2}$ = 0.02]. The HSA group showed greater orienting (57 ms) and alerting (52 ms) levels than the LSA group (orienting = 47 ms; alerting = 45 ms). However, the interference index was similar in both groups (HSA = 92 ms, LSA = 93 ms; *F* < 1).

The affective manipulation × network interaction was also significant in this analysis [*F*(2, 296) = 15.08, *MSE* = 677.57, *p* < 0.001, $\eta_{p}^{2}$ = 0.09]. Consistent with previous findings, when the emotionality of stimuli was manipulated, the alerting index was larger compared to when it was not manipulated (60 ms vs. 38 ms) [*F*(1, 150) = 28.65, *MSE* = 619.36, *p* < 0.001, $\eta_{p}^{2}$ = 0.16]. Conversely, the orienting network index was smaller when the emotionality of alerting signals was manipulated than when it was not (46 vs. 57 ms) [*F*(1, 150) = 10.95, *MSE* = 485.28, *p* = 0.003, $\eta_{p}^{2}$ = 0.05]. Furthermore, the interference index tended to be significantly greater when emotionality of stimuli was manipulated compared to when it was not manipulated (97 vs. 88 ms) [*F*(1, 150) = 3.07, *MSE* = 933.51, *p* < 0.081, $\eta_{p}^{2}$ = 0.02].

**Figure 3:**
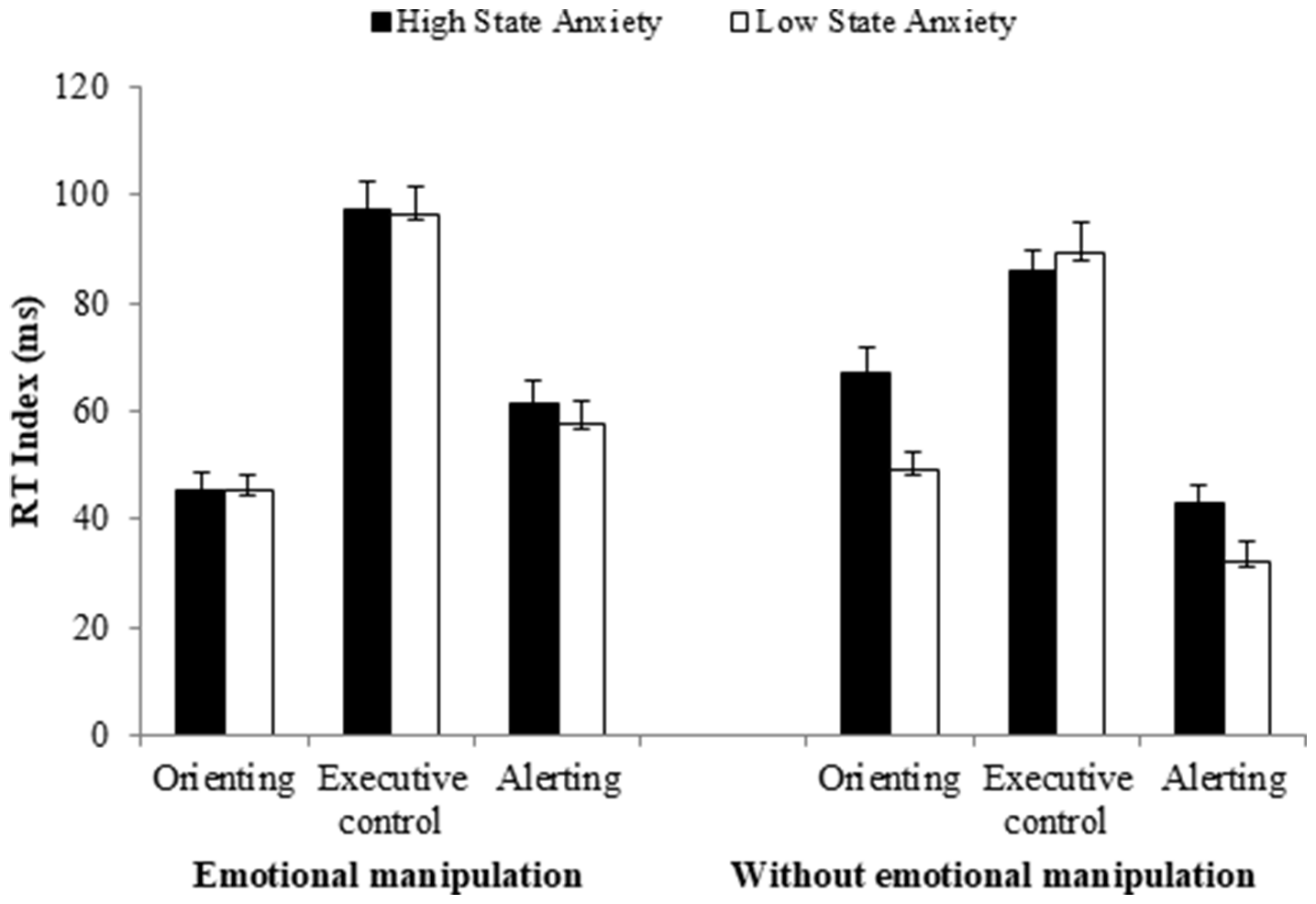
Reaction time (RT) indices of the three attentional networks as a function of participant group (high vs. low state-anxiety) for experiments with and without emotional manipulations of the alerting signal. Error bars represent standard errors of the mean.

The group × affective manipulation interaction was not significant [*F*(1, 148) = 1.86, *MSE* = 663.11, *p* = 0.174, $\eta_{p}^{2}$ = 0.01], and neither was the group × network × affective manipulation interaction [*F*(2, 296) = 1.35, *MSE* = 677.57, *p* = 0.259, $\eta_{p}^{2}$ = 0.00]. However, as illustrated in Figure 3, it is evident that manipulating the emotionality of alerting signals eliminates the group differences in the functioning of the attentional networks.

Regarding executive control, the level of interference increased slightly when the emotionality of stimuli was manipulated, compared to when only neutral signals were presented (97 vs. 88 ms). However, the difference between these two conditions was not significant [*F*(1, 150) = 3.07, *MSE* = 933.51, *p* = 0.081, $\eta_{p}^{2}$ = 0.02]. There were no differences between high and low SA in any case (*Fs* < 1).

In relation to alertness, an over-functioning effect was observed when affective signals were included, compared to when only a neutral tone was presented [*F*(1, 510) = 28.65, *MSE* = 619.36, *p* < 0.001, $\eta_{p}^{2}$ = 0.16]. This effect was noted in both groups, although it was greater in the LSA (affective signals = 58 ms; neutral = 32 ms) [*F*(1, 76) = 19.13, *MSE* = 661.79, *p* < 0.001, $\eta_{p}^{2}$ = 0.20,] than in the HSA group (affective signals = 61 ms; neutral = 44 ms) [*F*(1, 71) = 7.39, *MSE* = 565.85, *p* = 0.008, $\eta_{p}^{2}$ = 0.09]. The enhanced effect observed in the HSA group compared to the LSA group in experiments without emotional manipulation [*F*(1, 61) = 6.92, *MSE* = 369.82, *p* = 0.010, $\eta_{p}^{2}$ = 0.10], is eliminated when emotionality is manipulated (*F* < 1), as both groups show a larger alertness effect. It seems that the manipulation of emotionality already induces a state that consequently enhances the overall alertness effect.

Regarding the orienting network, a decrease was observed in both groups when the emotionality of stimuli was manipulated, compared to conditions in which only neutral signals were presented [*F*(1, 150) = 8.71, *MSE* = 485.28, *p* = 0.003, $\eta_{p}^{2}$ = 0.05]. This reduction was more pronounced in the HSA group (affective signals = 46 ms; neutral signals = 65 ms) [*F*(1, 71) = 9.95, *MSE* = 523.01, *p* = 0.002, $\eta_{p}^{2}$ = 0.12], than in the LSA group (affective signals = 45 ms; neutral signals = 50 ms; *F* < 1). The HSA group presented a significantly larger effect compared to the LSA group with neutral stimuli conditions [*F*(1, 61) = 4.90, *MSE* = 578.96, *p* = 0.030, $\eta_{p}^{2}$ = 0.07], an effect that was eliminated by introducing emotional stimuli (*F* < 1).

In summary, these analyses highlight that the manipulation of affective material as an alerting signal seems to exert three main effects on the functioning of the attention networks: the alertness effect is increased, the interference effect is increased, and the orienting effect is decreased in the presence of affective information. The magnitude of these effects varies across different anxiety groups, resulting in the elimination of previously observed differences in neutral task conditions by introducing the emotional manipulation. That is, the usual larger interference observed for the HTA disappears (the LTA group behaves as the HTA group in the presence of emotional stimuli), as it does the larger alertness and orienting effects observed for the HSA group. Nevertheless, we should be cautious with the interpretation of this pattern of results, as the analyses were performed *ad hoc* without *a priori* hypotheses. Furthermore, no significant interaction was observed between the manipulation of emotionality and the effects of anxiety groups, although the expected effects were only observed when no emotionality was manipulated, perhaps due to a lack of statistical power (note that the used sample size was not computed to test this specific interaction). Therefore, future research with a priori computed sample sizes should test the hypothesis that the effects of anxiety on the attentional networks significantly depend on the manipulation of emotionality.

## 5 General discussion

We report two experiments investigating the impact of affective alerting signals on the functioning of the attentional networks in individuals with high vs. low trait or state anxiety. We modified the ANT-I by introducing neutral or negative faces as alerting signals, presented alone or with a neutral tone. The results, along with additional analyses from eight experiments involving various anxiety groups, yield three key implications.

First, the alerting network shows greater reactivity when affective alerting signals are used, regardless of whether they are visual or bimodal. However, once affective stimuli are introduced, this increased alertness is observed for both neutral and affective stimuli. This suggests that alertness is sensitive to affective manipulations but not on a trial-to-trial basis. These findings align with previous studies using emotional sounds as alerting signals (Pacheco-Unguetti et al., 2009). Notably, the over-functioning (i.e., increased alertness effects) observed in HSA participants in neutral conditions (Pacheco-Unguetti et al., 2010, Experiment-2) was also present in LSA participants when emotional stimuli were introduced, equalizing alerting levels across groups.

Second, the interference effect is greater with affective stimuli. The established effect of alertness on the executive control network (larger interference when alertness is high) is even stronger with affective alerting signals. This influence eliminates the differences previously found between high and low TA (Pacheco-Unguetti et al., 2010, Experiment-1) due to a larger increase in interference in LTA groups. For SA groups, enhanced alertness generally impairs the executive control network, even though SA was not associated with higher interference in neutral conditions.

Third, the orienting network effect is diminished with affective alerting signals, especially in HSA groups. This reduction eliminates the previously observed differences between high and low SA groups (greater orienting effects in HSA) without affective manipulations (Pacheco-Unguetti et al., 2010, Experiment-2). The relationship between alerting and orienting networks is complex. While orienting is positively related to alerting under non-affective conditions (i.e., a larger orienting effect is observed after a warning signal), its functioning can be enhanced or reduced depending on the emotional state and affective manipulation of alerting signals. Introducing affective information as alerting signals seems to concentrate reactivity toward alerting stimuli, diminishing spatial orienting even among individuals with induced SA.

Overall, our findings demonstrate that the alerting network is not transiently affected by the valence of visual or auditory stimuli. This suggests that all alerting stimuli inherently increase emotional load, so alertness is not further increased by the emotionality of the alerting stimulus. However, the alerting network’s functioning is enhanced in the context of affective stimuli, either by introducing complex affective stimuli within the task or by inducing a negative emotional state prior to task performance. This could explain why the larger alerting effect observed after inducing an anxiety state without emotional manipulation of the alerting signal is not observed when the alerting signal includes affective information. Thus, the alerting network reacts to the basic sensory attributes of stimuli, which may signal danger or the need for preparation, rather than their emotional valence.

To better understand this, it is worth considering the differentiation between phasic alertness (manipulated and measured in our studies) and tonic alertness or vigilance. Although these two processes are related to a large extent, they have some key differences that may help clarify our results (Klösch et al., 2022; Luna et al., 2018; van Schie et al., 2021). Phasic alertness refers to a state of generalized readiness following a warning stimulus (Posner, 1980; Raz and Buhle, 2006; Sturm and Willmes, 2001), while vigilance represents a focused readiness to detect and respond over an extended period. Phasic alertness does not require deep processing of warning stimuli in terms of affective valence, while vigilance involves more deliberate and prolonged processing (Brown and Bowman, 2002).

The lack of differences in alerting regarding trial-by-trial valence manipulations in our experiments is likely due to its function of unspecific preparation for sensory information. This function can be carried out without profound affective processing and may be determined only by the relevance of stimuli to a future target response. Some studies have attempted to explain the influence of affective manipulations on attentional networks by considering the task relevancy of these manipulations (Cohen et al., 2011). From this perspective, all alerting signals, both affective and neutral, are inherently relevant because they anticipate upcoming information that requires processing.

This target preparation, once alertness is enhanced by affective signals, could explain the reduction in orienting and the increase in interference effects. When alertness is highly activated, we prepare for rapid reactions to incoming information, allowing for broader processing of both the target and distracting stimuli. Consequently, the enhanced rapid reaction to the target minimizes orienting effects, while increased reactivity to distracters increases interference. Weinbach and Henik (2011, 2012) suggest that alertness influences executive control by promoting a global processing style, increasing the accessibility of both relevant and irrelevant task stimuli. In our experiments, affective manipulations of alerting signals may have strengthened the alertness influence on the control network, leading to greater interference from distracting stimuli.

Importantly, this effect is observed across all anxiety groups, indicating that affective information renders all participants equal regarding their control of interference. This is particularly relevant given that high anxiety is typically related to an impaired ability to manage distracters, even when these are emotionally neutral (Berggren and Derakshan, 2013, 2014; Bishop, 2009; Pacheco-Unguetti et al., 2010). Therefore, individuals with low anxiety, when exposed to affective stimuli, behave similarly to those with high anxiety in neutral conditions. Highly anxious people react as if there were always emotional information present, rather than selectively responding to the nature of the current information and context.

In summary, including negative affective signals as alerting cues does not have a significantly greater trial-by-trial impact on the alerting network compared to signals with another affective valence, although its overall effects are larger compared with a neutral alerting tone. Furthermore, these affective signals clearly influence the overall functioning of the orienting and executive control networks. Presenting affective alerting signals before the initiation of the orienting process reduces the effects of the orienting network on target processing and impairs the executive control network. The lack of studies focusing on specific affective manipulations of phasic alertness, along with the multiple constructs and neural systems underlying the alerting system (i.e., Berridge and Waterhouse, 2003; Oken et al., 2006; Posner, 2008; Yanaka et al., 2010), has made it difficult to clarify the role of affective valence on this attentional network and its impact on other networks.

Additionally, individual differences make the study of these relationships more complex. The pattern of results suggests that anxious participants are not significantly more disturbed regarding conflict resolution when irrelevant affective information is presented. This may be explained by the tendency of anxious participants to apply a lax criterion in differentiating between neutral and affective contexts in conflict situations, treating all conflict situations as threatening. Conversely, participants with low anxiety perform better in neutral situations but show difficulties with conflict resolution in the presence of affective information.

## Data Availability

The raw data supporting the conclusions of this article will be made available by the authors, without undue reservation.

## Footnotes

^1^Apart from the data of the current paper, data from previous papers were included, taken from Pacheco-Unguetti et al. (2009, 2010), and from an Erp study in which participants were selected on the base of their scores in trait-anxiety. We did not include data from Pacheco-Unguetti et al. (2011) because in this study the great majority of patients with anxiety disorders reported a high level of both state and trait-anxiety. In all the mentioned experiments (both published and unpublished), our selection criteria were consistent. Participants scoring ≥ 34 (80th percentile) on the trait-anxiety measure were classified into the Hta group, while those scoring ≤ 14 (15th percentile) were placed in the Lta group. Participants with medium-to-low trait-anxiety levels (percentiles between 25 and 35) were further included into either the high or low Sa groups. The Spanish version of the Stai includes 20 items, each scored from 0 to 3, so that the total varies from 0 to 60, rather than from 20 to 80, as in the English version.
